# What helps patients access web-based services in primary care? Free-text analysis of patient responses to the Di-Facto questionnaire

**DOI:** 10.1186/s12875-023-02257-5

**Published:** 2024-01-10

**Authors:** Nada Khan, Emma Pitchforth, Rachel Winder, Gary Abel, Christopher E. Clark, Emma Cockcroft, John Campbell

**Affiliations:** Exeter Collaboration for Academic Primary Care, College of Medicine and Health, University of Exeter, Exeter, UK

**Keywords:** Primary health care, Digital divide, Internet use

## Abstract

**Background:**

The National Health Service (NHS) and general practice are increasingly adopting digital services. These services can impact both positively and negatively upon patient experiences, and access to digital services is not equal amongst all groups. Within a wider project examining digital facilitation (the Di-Facto study) our team conducted a patient survey amongst English primary care practices aiming to investigate patient views of what supports uptake and use of web-based services. This paper reports on the analysis of the free-text responses from the patient survey.

**Methods:**

The Di-Facto patient survey was distributed to practices in eight clinical commissioning groups (CCGs) in England between 2021–2022. We examined free-text responses to two questions relating to access to primary care web-based and support for web-based services. We used qualitative reflexive thematic analysis based on a six-stage process to analyse responses.

**Results:**

Of the 3051 patients who responded to the Di-Facto survey, 2246 provided a free-text response. We present our findings in two major themes: systems and structures and their impact on use of web-based services, and ‘what works for me’, a description of how respondents described what worked, or did not work in terms of their interactions with web-based services. Respondents described how the technology, such as poor practice website design, confusion over multiple digital apps, data security and concerns about eConsultation offerings impacted on use of web-based services. Respondents described practice level barriers, such as a lack of or inconsistent provision, which prevented optimal use of web-based services. Respondents described personal and technical barriers that impacted on their use of digital services, and described which web-based services worked well for them. Respondents felt that web-based services were not a replacement for face-to-face interactions with a doctor.

**Conclusions:**

This analysis of free-text responses from a large patient survey highlights the system, practice, and person level barriers and facilitators to use of digital services in primary care. With an increasing push towards digital solutions in NHS primary care, practices should consider the design, rollout and communication of their web-based services to support patient access.

## Background

The National Health Service (NHS) is moving towards a digital age, with policy aiming to increase use of digital tools to transform and improve the experience of care from both the patient and clinician’s perspective. In 2019, the NHS Long Term Plan set out a programme which aimed to continue digitising appointments and prescriptions, increase use of a digital NHS ‘front door’ through the NHS App, and offer a digital-first approach to primary care to facilitate ‘fast and convenient’ access to primary care [1, 2]. These changes have been accelerated by the Covid-19 pandemic, with a dramatic increase in patient access to primary care and use of digital services since 2020 [3]. The web-based services made available to patients in the NHS vary by practice but typically involve means to book appointments, order repeat prescriptions, contact their GP or other healthcare professional for advice and to access their health record. Modes of access also vary by practice, but typically involve use of either individual practice websites or the NHS app.

Digital services, which are also increasingly used across financial and commercial sectors, aim to benefit patients in terms of patient choice, increased convenience and improved ease of access, and clinicians by improving triage systems and offering more efficient service delivery. However, these services can impact both positively and negatively upon the patient experience of using primary care [4, 5]. There is a strong deprivation gradient in both awareness and use of some digital services. Differential access to digital services can widen inequalities, with poorer access amongst low socio-economic groups, non-white ethnicities and amongst older age groups [6]. The Di-Facto programme aimed to investigate the potential for digital facilitation, defined as ‘a range of processes, procedures, and personnel which seeks to support NHS patients in their uptake and use of online services’ [7]. The wider Di-Facto study involved four elements, including a scoping review of digital facilitation approaches to improve access to online services, a survey of general practice staff to explore which online services were being used and supported by their practice, and a qualitative exploration of digital facilitation incorporating focussed ethnographic case studies in general practices and an interview study with key stakeholders. Part of this programme of research also involved a patient survey to examine patient awareness and experiences of digital facilitation and patient awareness and use of online services [8]. This paper aims to investigate patient experiences of using web-based services in primary care through a qualitative thematic analysis of free-text responses from the Di-Facto patient survey.

## Methods

### Questionnaire

The Di-Facto patient questionnaire was developed iteratively over a series of meetings with the research team and the project’s patient advisory group (PAG) and was informed by previous research and the findings of a literature review [9]. The questionnaire included a total of 30 questions including the two free-text questions, and focussed on online services offered from the respondent’s GP practice and how helpful any support was from the practice [8]. Practices were randomly selected from eight Clinical Commissioning Groups (CCGs: Devon, Birmingham and Solihull, South Warwickshire, Coventry and Rugby, Cambridgeshire and Peterborough, East and North Hertfordshire, Enfield and Haringey) and first completed a practice-level survey. Additional practices were selected to oversample from those serving deprived populations. Participating practices were asked to produce a random sample of patients aged 16 and over after excluding patients with severe mental illness, recent bereavement, or those not able to give informed consent. To ensure inclusion of deprived communities and issues relating to systematic differences in predicted response rates, practices serving highly deprived populations were asked to select the first 285 patients, practices serving populations of medium deprivation to select 220 and those serving the least deprived population 150 patients, to receive the questionnaire [10]. Questionnaires were sent to the practice’s patient sample by post. Participants could either return the survey in the supplied freepost envelope or complete the survey online. The first mailout was completed in September 2021, the last in May 2022, and patient responses were accepted until July 2022.

### Research approach, theoretical perspective and positionality

We used a reflexive thematic analysis where we acknowledged that the analysis was situated within an interactive process that reflected not only the data, but the positionality of analysts and research team and the context of the research within the wider Di-Facto study [11]. Analysis of the free-text responses was led by one member of the team, NK, who is an academic GP registrar, alongside EP, a non-clinical researcher. NK’s perspectives as a primary care clinician may have influenced coding, themes and interpretation. To try to account for potentially disciplinary biases initial codes and developing themes were checked by EP to ensure that the data itself, and not the preconceived notions of the analysts, informed the analysis. The authorship group brought multidisciplinary perspectives as part of the wider DiFacto research team.

### Analysis

We examined responses to the two free-text questions from the Di-Facto survey (Table 1). One member of staff entered data from paper surveys manually and a second member of staff double-checked the first 100 responses.

**Table 1:** Free-text questions in the Di-Facto questionnaire

Q16 What can the practice do to help you access the online services?
Q17 Is there anything else you would like to add about online services and the support at the practice to help you use them?

This study used a reflexive thematic analysis approach to identify and analyse patterns and themes in the dataset to explore the views of the respondents. This involved a six-stage process as detailed by Braun and Clarke, and as a reflexive analysis, reflects how the researchers conceptualised the data and is an interpretation of the responses [12]. One researcher (NK) initially read, and re-read, all the free-text responses to familiarize herself with the data and to note down initial ideas. NK then generated initial codes by reading all the responses and coding the substantial responses. A subset of the data was read and coded by EP and both NK and EP met to discuss the initial coding. Data relating to both questions were considered together. Throughout the process, codes and higher-level categories were re-reviewed and re-coded, merged and collapsed as an iterative process. NK then collated codes into potential themes, and reviewed the themes in relation to the coded data at the individual level and across the dataset to develop a visual thematic map of the developing analysis. EP reviewed the coding and the developing themes at this stage. The themes were then refined based on the initial dataset and re-organised into the final overarching themes. Relevant quotations were selected to illustrate the generated themes. Quotes are displayed as they were written by the respondent, and have not been amended for spelling or grammatical errors. We used NVivo 12 software (QSR International Pty Ltd) to manage data.

### Patient and Public Involvement and Engagement (PPIE)

The initial codes and early groupings of the codes were presented to the Di-Facto study PAG, who have been involved throughout the wider study, for discussion. The group comprises nine patients with experience of using primary care services. During an online workshop, the PAG provided feedback on the code descriptions and headings, and suggested coding structures and collation based on their experiences of using digital services in primary care. Their feedback helped to guide the groupings of codes within each theme, for instance, the codes that did not specifically relate to digital facilitation (I want a human, personal barriers) were amalgamated into the theme ‘Why digital health technology does not work for me’.

## Results

Sixty-two practices invited 12,822 patients to respond to the patient survey. 3051 patients returned questionnaires (598 online, 2453 paper) and in total 2246 free text responses were provided across the two questions (Table 2). Of responders to the main patient survey, 57% of the sample was female, 45% were 65 or older, the vast majority described themselves as white (93%), 9% reported that English was not their first language, 44% were working either full time or part time, and 43% were retired [8]. Responses ranged in length from single words to multiple sentences. Analysis of the main questionnaire will be reported separately.

**Table 2:** Breakdown of responses to the free-text questions

	Q16 free text responses	Q17 free text responses	**Total**
Paper survey	905	695	1600
Online survey	396	250	646
**Total**	1301	945	2246

We report the results from the free-text analysis in two major themes: systems and structures and their impact on use of web-based services, and ‘what works for me’, a description of how respondents described what worked, or did not work in terms of their interactions with web-based services.

### Systems and structures

**Fig. 1:**
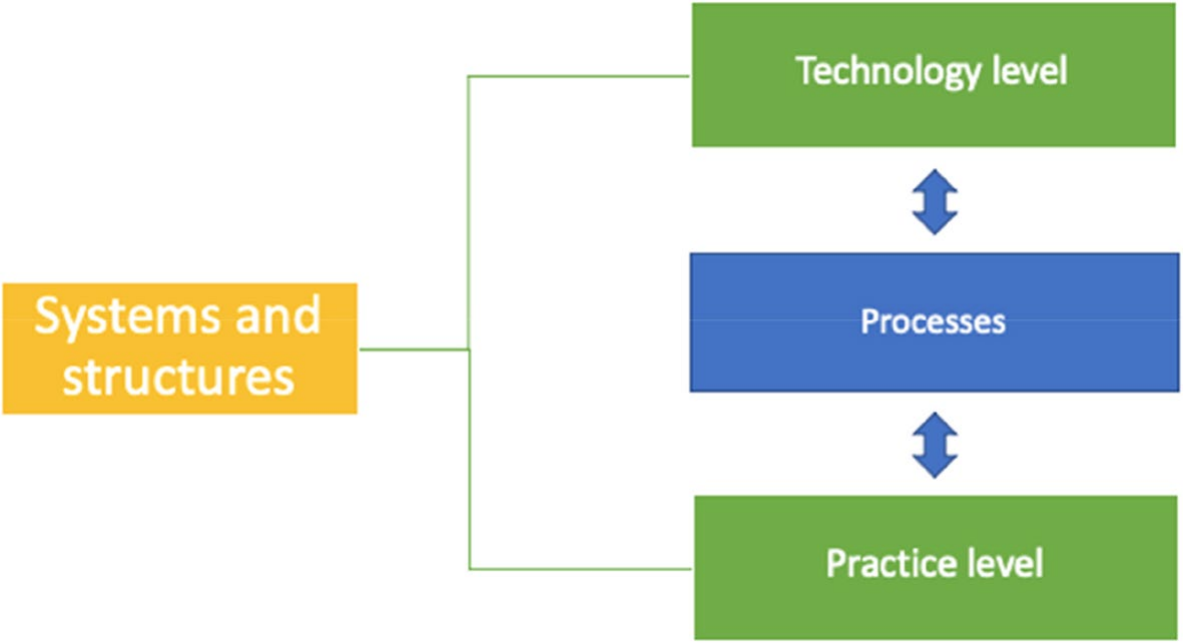
Systems and structures and use of web-based services

Respondents described several areas where the systems and structures of health service technology impacted on their use of web-based services. This theme is separated into two sub-categories relating to systems and structures at the technology level and the practice level (Fig. 1). Respondents also described the interplaying processes between the technology and primary care practice systems and structures.

#### Technology level

Survey respondents described how technology issues, including perceptions about practice websites, information about services on offer, eConsultation services and concerns about data security affected their access and use of services offered by their practice. Respondents frequently mentioned that practice websites were difficult to navigate, out of date and confusing. Information about which web-based services were available was not always clearly highlighted on the home page, which meant that these services were hard to identify. Respondents disliked ‘busy’ websites that were ‘too complicated’ or required them to view several pages to find information, for instance, this respondent wanted her practice to ‘make the website simpler to use instead of multiple click through different pages to get to where you want to go.’ (Female, aged 65–74, online response). A clearly organised website was viewed as especially important for people who were less confident using web-based services, ‘It needs to be a tad more user friendly as there is a lot of info, icons and links everywhere which can make it confusing to know where to search for things. Especially if you are trying to teach someone who is less fluent in technology.’ (Female, aged 25–34, online response).

Having an up-to-date and clear website was important in terms of helping users identify available web-based services. This respondent describes that her practice needs to ‘update their website so all available online services are clear… I need to be confident that the information displayed on the website is up to date’ (Female, aged 55–64, online response).

Some participants described how NHS services seemed to be disjointed in terms of multiple and overlapping web-based services. Respondents felt that this was ‘confusing’ (Male, aged 35–44, online response) and wanted services to be combined or ‘simplified’. For instance, one participant who used two web-based services, MyChart (to access test results) and Patient Access (to order repeat prescriptions) thought it would ‘make sense to consolidate apps.’ (Male, aged 45–54, online response). When there were multiple possible ways to contact the GP surgery, it was unclear which to use for each different request. This respondent felt that the only difficulty she had experienced was ‘knowing which service to use for which query’ and wanted her practice to ‘indicate more clearly when to use the various options: email, online appointment system, NHS App, eConsult.’ (Female, aged 45–54, online response).

Data safety and security were seen as important issues specific to use of digital services. Respondents raised concerns about web-based services and confidentiality of health data, data security, and use of data by other third-party organisations. Respondents wanted to be assured that their data was being kept confidential. One respondent wrote, ‘Confidentiality is v.important – would need to feel more secure about my information online’ (Female, aged 45–54, paper response). A few respondents described how publicity about online scams led to a distrust of online health services. One respondent felt that it ‘would be good overall to understand security of systems – not specific to this practise but as a whole’ (Female, aged 35–44, online response), while others wanted assurance, or ‘convincing’ that their personal information was safe.

Respondents wanted to know that their health care data was not being used for any other purposes. This respondent described how ‘all IT systems must be easy to use, robust and most importantly performant and trustworthy. The maintenance and use of data collected online must be transparent (and this data must not be used/sold for marketing purposes under any circumstances)’ (Female, aged 55–64, online response).

Respondents described their experiences and interactions with eConsultation services. NHS eConsultation services allow practices to conduct digital triage, and patients can submit clinical queries and requests for prescriptions. A few respondents felt that the eConsultation service had worked well for them, and was ‘good’ or ‘excellent’. One participant described how having a quick response from an efficient GP worked well for them as ‘the GP is really efficient and responds to the e-consult very quickly. Often I do not require a phone call and they can text me the information which works well for me. For example, that my prescription has been done’ (Female, aged 16–24).

More commonly, respondents raised concerns and frustrations from their interactions with eConsultation services. Several respondents were frustrated that the service was not always available for use or that eConsultations were only available to submit within office hours. One respondent described how ‘The eConsult form ‘window to complete’ is about 3 min per day’ and felt that ‘this could certainly be improved’ (Female, aged 35–44, paper response). One respondent described how the time windows for submitting eConsultations meant that the service was not accessible for those who work full-time, ‘They only open the eConsult during opening hours, which doesn’t work if you work full time and have weekend off. This makes online services inaccessible for some.’ (Female, aged 16–24, online response).

Other respondents described their negative experiences completing eConsultations. Most eConsultation software has a series of questions to triage emergency medical situations that need immediate emergency care. These questions were perceived as ‘stupid’, ‘unnecessary’, ‘irrelevant’ and ‘repetitive’. One respondent stated that ‘Econsult asks too many stupid questions. Are you breathing? Are you bleeding uncontrollably? Has your heart stopped? It makes a great service time consuming, frustrating and tedious.’ (Female, aged 45–54, online response). The number of questions on the eConsult were especially difficult to fill in for respondents with mental or physical health issues, as questions increased stress, and were ‘not good for someone who has mental health issues’ (Female, aged 55–64, paper response). It was often unclear when a call back would occur following an eConsult. One respondent suggested that it would be useful to have a specified time for the call back, ‘When you book online it tells you someone will be in touch in the next 2 days but it should give you a day + time, so you can sort time for this.’ (Female, aged 55–64, paper response).

Finally, some respondents described how it was unclear who was looking at submitted online requests, and wanted reassurance that a health professional was acting upon the inquiry. Uncertainty about who was reviewing eConsultations meant that other respondents were less likely to use the service, especially for ‘personal matters’.

#### Practice level

Aside from issues with the technology of web-based services, some respondents described practice level barriers and problems at their GP surgery which prevented optimal use of web-based services. These included withdrawing, or not providing web-based services at a practice level.

A few respondents described how their practice did not offer web-based services, or where these services were offered, their use was discouraged. For instance, practices had offered services such as web-based appointment booking which were withdrawn over the Covid pandemic, and several respondents felt that this would be a useful service to offer again.

Respondents used the free-text responses to describe their overall opinion about the state of general practice, and ‘broken’ GP services. Some of these issues were not specifically related to web-based services. Respondents described how it was difficult to get an appointment with their GP or to get in touch with anyone at the practice by phone. A few respondents felt that web-based services might help save time booking appointments, as one respondent described how they would ‘be happy to make appts online rather than spending hours on the phone waiting to be answered’ (Female, aged 65–74, paper response) and another stated that ‘this survey has prompted me to look into online booking further. I currently book all appointments over the phone which is often a lengthy and frustrating process.’ (Male, aged 55–64, online response). Others felt that technology was no replacement for people and felt that their practice needed to employ more staff. ‘More staff at the surgery would be more helpful rather than replacing people with technology’ (Female, aged 35–44, paper response).

#### Processes between technology and practices

One issue commonly raised by survey participants was the process for registering their details to access the web-based services provided by their practice. Some patients described the registration process as ‘difficult’ and wanted the registration process to be simpler and quicker, with login codes sent to them electronically or by post. Some practices required patients to come into the practice to show personal identification or to get passwords, which respondents found difficult especially during the Covid pandemic lockdowns. One respondent wanted her practice to ‘make the registration process easier—it involved in person documentation of multiple addresses and that kind of defeated the point of wanting online access’ (Female, aged 25–34, online response).

The registration process was not straightforward for some participants even after they received access passcodes for web-based services. A few patients described receiving verification codes that didn’t work, or not having the right access credentials when attempting to register for or use services. The registration process was perceived as ‘long winded’ and several respondents wanted the process to be ‘easier’.

Some respondents in this study highlighted the difficulties in substituting real-time interaction with an asynchronous technological interface. Respondents described it being unclear if web-based forms, e-consults and emails had been read or actioned by the practice. To mitigate this uncertainty, a few respondents wanted their practice to reply to submitted eConsultations, acknowledge email receipt, or confirm that requests such as prescription orders had been received and actioned. This respondent, who used web-based services, stated how she was ‘very comfortable using such services (as is preferred by the gp team for routine/non urgent enquiries) however there have been occasions when I have sent emails requesting an eConsult or test results and the email was not responded to or acknowledged. It would be useful to get a acknowledgment of receipt of email’ (Female, aged 45–54, online response). Generally, it was unclear when and if a response would be made to an online request. One respondent described how ‘you never know if or when you are going to get a response. At least a conversation is interactive’ (Male, aged 25–34, online response).

### What works for me

**Fig. 2:**
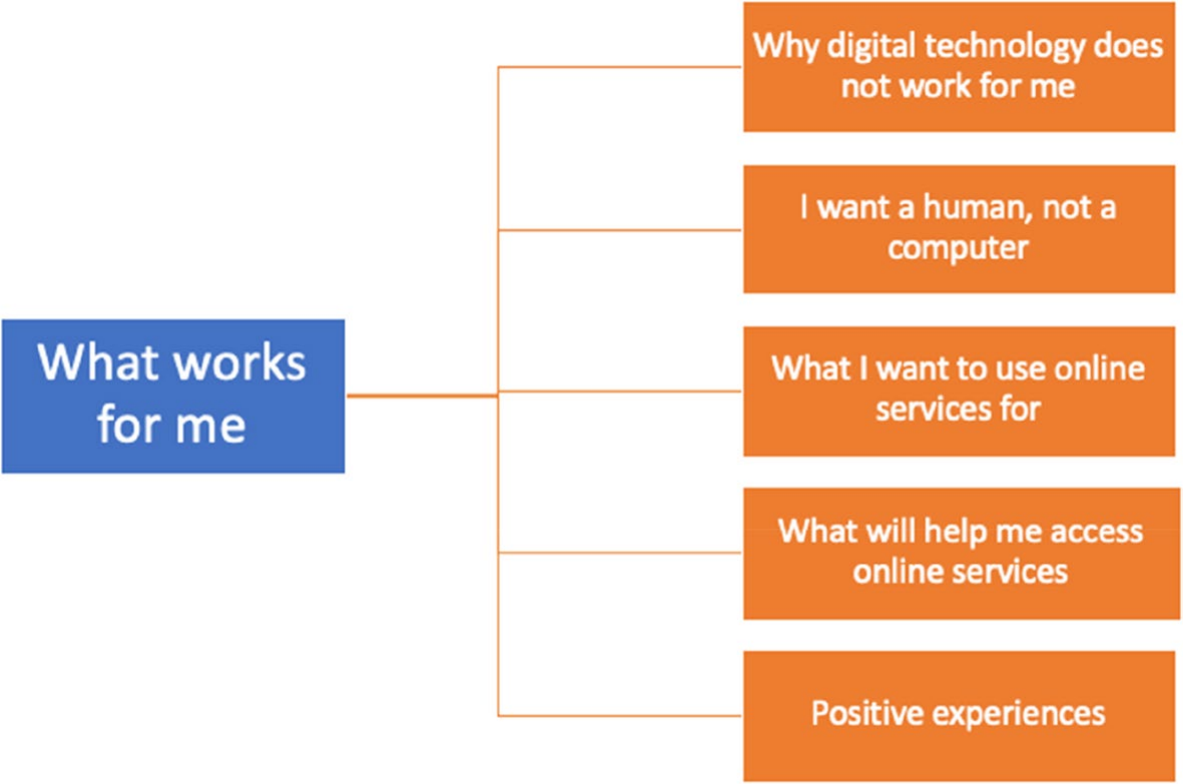
Patient views of ‘what works for me’ and web-based services

This overarching theme describes what respondents described as what worked well for them in practice relating to web-based services, and is described in detail in five subthemes (Fig. 2).

#### Why digital health technology does not work for me

Respondents commonly described why personal barriers, including age, poor eyesight, low English literacy and learning disabilities meant that they found using web-based services difficult. One respondent described how they were ‘getting old and don’t want to be bothered and I’m almost as blind as a bat’ (Female, aged 65–74, paper response). Poor access to web-based services was viewed as isolating by some respondents, such as this respondent, ‘as an 88 year old technophobe I feel more and more isolated by “progress” in the NHS as so many of my generation.’ (Female, aged 85 + , paper response). A few respondents highlighted that it was difficult to use web-based services whilst feeling unwell, or that their mental health meant that they felt more reassured talking to someone rather than engaging with an online service. This respondent wrote ‘I hate computers + want a personal service. Computers make me anxious and don’t help my mental health’ (Male, aged 55–64, paper response).

Other respondents described technical barriers, including no access to a smartphone or computer, no internet, poor internet or phone reception, or low IT literacy as limiting their access to web-based services. This respondent felt that ‘the problem lies more with me. I don't have up to date computer or mobile phone, which makes accessing very difficult.’ (Female, aged 65–74, paper response).

Some respondents described that they were just ‘not interested in online services’ and didn’t want to use them. Many of these comments were brief, with a few respondents writing, ‘I do not wish to use online services’ or ‘I am not interested in online services’, with no specific reason given.

#### I want a human, not a computer

Several respondents detailed how they did not want to use web-based services because they were no replacement for a meaningful face-to-face interaction with their own doctor, a ‘real person’. Some wrote about wanting to see ‘a human’ instead of a ‘robot’ or computer, for instance, stating ‘I want a doctor, not a computer’ (Female, aged 55–64, paper response), and ‘I want to speak with a real human. Online is too impersonal. I have never used their online services. I want to speak with a real human.’ (Male, Age 45–54, online response).

Some respondents perceived that during the Covid pandemic’GPs seem to have “disappeared”’ (Female, aged 55–64, online response) and were unhappy with the shift away from face-to-face appointments following the Covid lockdown. Respondents described wanting to ‘change’ back to face-to-face appointments and felt that Covid was used as an ‘excuse’. One respondent described how Covid had impacted on his ability to speak to his doctor in person and how this affected trust in his care, ‘I associate on-line services with attempts by my GPs to avoid face to face consultations—and delay. That is a step backwards in the service level of the past. Over the Covid pandemic our surgery made it close to impossible to see a doctor face to face. This has destroyed trust in the commitment to care doctors should offer’ (Male, aged 75–84, online response).

One respondent described how web-based services were ‘overly complicated’ compared to speaking to a ‘human’, a sentiment that was mirrored by respondents describing how it ‘tends to be quicker to call’ (Female, aged 25–34, paper response), and that ‘all online services are complex and time consuming a telephone conversation is always quicker’ (Female, aged 65–74, paper response). Respondents acknowledged that phoning the practice was problematic given long phone messages and the length of time it took to speak to someone.

#### What I want to use web-based services for

Respondents described which web-based services they wanted to use, including booking appointments online, ordering prescriptions, accessing their medical records and viewing test results. Some practices offered online appointment booking, but suspended the online booking of appointments during the Covid pandemic which was seen by one respondent as a ‘huge backwards step’ (Female, aged 45–54, online response). In general, respondents felt it would be ‘nice’ to be able to book appointments online again instead of ‘spending hours on the phone’ (Female, aged 65–74, paper response).

Some respondents only used web-based services to order repeat prescriptions online, a service that was described as ‘good’ and ‘efficient’ by most using it, however the process was not always straightforward and described as ‘tortuous and constantly changing’ (Male, aged 65–74, paper response) and ‘awful’ (Male, aged 55–64, paper response) by others.

#### What will help me access web-based services?

Respondents suggested that promotion of web-based services, information about what was on offer, training and support from the practice would help them access web-based services in the future. Suggestions to increase visibility included sending letters and leaflets, emails or texts about what online services were available to use. One respondent described how ‘I think the practice can help by sending out emails or messages that inform patients of the online services available to them as I did not know of these online services until I received this research letter.’ (Male, aged 16–24, online response). Several respondents wanted specific training in the form of tutorials, videos, online and in-person workshops on how to register and use online services. Respondents suggested teaching sessions and ‘step by step’ guides on what services were available and how to use them.

#### Web-based services work for me—positive experiences

In contrast to negative experiences, many respondents described positive experiences of the web-based services offered by their practice. Web-based services were described as better than traditional alternatives, for instance, instead of having to phone the practice and ‘wait in a queue’ for an appointment or results. Several respondents felt that web-based services were quick, efficient and convenient. Certain services were highlighted as working particularly well for ordering repeat medications and viewing test results. Respondents felt that web-based services were suitable for non-urgent issues. This respondent described how ‘when I have used eConsult before, it was very helpful and probably easier than having to call and wait in a queue. It does feel a lot more impersonal and it’s harder to communicate when not actually talking to someone, but it's a good alternative for less serious issues’ (Male, aged 16–24, online response).

## Discussion

### Main findings

This paper reports the analysis of the free-text responses to a large questionnaire of patient experiences of digital facilitation in primary care. In a period where COVID-19 accelerated the use of digital primary care services, the views and experiences of patients are important to capture. We make a number of practical implications, based on the findings, that can inform the expansion of digital services (within a hybrid approach) and future efforts to support and enable patients to be able to use these services. In this study, respondents described difficulties using their practice website and eConsultation services, and highlighted the importance of up-to-date information, training and clear signposting about web-based services. Personal and technical barriers meant that not all respondents wanted, or were able, to use web-based services. For some, the move towards digital solutions was viewed as an impersonal service compared to face-to-face interactions with a GP. Respondents also described advantages to the use of eConsultation services other web-based services for certain interactions with their GP, including ordering medication and viewing their medical records.

### Comparison with other literature

A recent systematic review of approaches to digital facilitation in primary care suggests that low knowledge of which web-based services are available is a significant barrier to their use in primary care [9]. Training and education, for instance, through web-based videos or in-person seminars, may promote the uptake and use of digital services [9]. Some of the respondents in our study described needing support to access online services, and previous research has suggested that patient-centred guidance and practice champions as ‘experts’ may increase initial uptake or support continued use of web-based services [13, 14]. Practice website design was frequently highlighted as a factor in how easily respondents were able to access information about available web-based services. Previous research from Scotland has found that most practice websites do not meet readability, design or accessibility recommendations, which may increase digital exclusion [15].

Important aspects highlighted by this work are the unintended and intended consequences of online services. Similar to other research, some intended consequences, such as improving access to care, were described positively by respondents who used services such as eConsultation services or online prescription requests [16]. However, online consultation tools can make communication difficult for others, for instance, the frustration felt by some of our participants when filling in structured questionnaires for eConsultation services was mirrored in a study of online consultations in English primary care [17]. The asynchronous nature of some online services, and the increasing roll-out of online consultation models can disadvantage digitally-excluded patients who may face the kinds of personal barriers highlighted by the respondents in our study [17, 18]. Digital exclusion also impacts specific geographical populations, such as rural dwellers, due to inferior broadband access [19]. Such aspects have also been highlighted in discussions with the project’s PAG. This work emphasises the importance of such issues and an awareness of unintended implications when scaling up online service provision or models of digital facilitation to support patients to be able to use such services.

Respondents in our study described concerns about data security, confidentiality, and use of their data by third-party organisations. Studies of patients and online records shows that concern about privacy is a major issue [20]. As in our analysis, some patients do see benefits to web-based services in primary care. A review of online access to patient records and online services highlighted some benefits, including improvements in patient safety and self-care as patients are able to view test results, manage their medications, and identify errors in their medication list [21].

### Strengths and weaknesses

There are several methodological issues to consider when analysing free-text comments from questionnaires. Free-text responses are unlikely to be representative of the surveyed population because only a minority write comments, and those who do are usually more articulate or have a negative comment to make. As the comments are unrepresentative and self-selected, any findings emerging from the free-text analysis are not generalisable to the study population. Unlike in-depth interviews, we were unable to clarify comments that are unclear [22]. To mitigate these issues, we used a focussed research question based on the free-text questions within the survey, aiming to investigate patient experiences of using web-based services in primary care, and engaged experienced qualitative researchers in the analyses. Additionally, we included all the free-text responses in analyses to ensure that all comments were considered. Free-text questions are often included in questionnaires, and as part of the data provided by the respondents, it is important to look at and collate these responses in a meaningful way.

The questionnaire was sent to patients during the Covid-19 pandemic when many practices changed the way appointments and online services were offered. From April 2020, practices in England were directed to offer a remote ‘total digital triage’ model, which led to a rapid acceleration in online and telephone triage [23, 24]. It is possible that some of the issues highlighted by respondents to this study, such as the inability to book appointments online, may be managed differently as practices and patients adapt to changes in the offering of web-based services. Finally, the scope of our work was broad – we were therefore unable to comment specifically on differences arising from varying modes of non face-to-face interactions with health services eg web based, telephone, asynchronous and synchronous web or chat interactions.

### Implications for practice

Web-based services are negatively and positively experienced by patients in primary care. Clear communication to their patients about web-based services should be a priority for primary care practices. Respondents in this study underlined the importance of clear, well signposted and up-to-date information about which web-based services were available. Uncertainty, inconsistencies in provision and unexplained changes to web-based services can raise concerns or questions for patients, but this could be addressed by routinely providing information to patients. Information to patients about data security, confidentiality may also increase trust in web-based services.

With increasing use of eConsultation services, potential benefits of these services are confounded by restriction in hours of availability. Practices should reconsider access to such services and when patients can submit queries, and provide clear information about how the submitted request or information will be dealt with, by whom, and when a patient should expect a response. Access to web-based services is hindered by complex registration procedures which need to be clearly explained and simplified to increase uptake. Multiple and duplicating web-based services and apps, especially across primary and secondary care services, are frustrating to patients. Amalgamating or streamlining the number of web-based services may be a challenge for practices, however, these are the drivers of poor experience for patients and should be addressed.

Some respondents felt that individual barriers, such as their age, their access to the internet or internet-enabled devices or their digital literacy meant that they were increasingly isolated by moves to digitise health services in the NHS. Thus the digital divide, with its risk for widening inequalities of access, needs to be overcome [25]. The Di-Facto programme aims to tackling this issue, but action is required at regional and national levels to level up access to digital medicine and reduce inequalities [26, 27].

## Conclusions

Meeting the needs of the public are important when designing and implementing web-based services in primary care given the increasing use of digital technology in the NHS. Future digital service design and delivery could focus on using a framework of digital facilitation to overcome barriers at the level of system and structures, and personal barriers which impact on patient access to web-based services.

## Data Availability

Study data may be made available to appropriate individuals on a case by case basis following an application to the Chief Investigator (Professor John Campbell), email address for contact via Ellie Kingsland (e.kingsland@exeter.ac.uk).

## Abbreviations

CCG: Clinical Commissioning Groups
GP: General Practitioner
NHS: National Health Services
PAG: Patient Advisory Group
PPIE: Patient and Public Involvement and Engagement
